# Lightweight Lung-nodule Detection Model Combined with Multidimensional Attention Convolution

**DOI:** 10.2174/0115734056310722241210055412

**Published:** 2025-01-02

**Authors:** He-He Huang, Yuetao Zhao, Sen-Yu Wei, Chen Zhao, Yu Shi, Yuan Li, Weijia Huang, Yifei Yang, Jianhua Xu

**Affiliations:** 1 Ocean College, Jiangsu University of Science and Technology, Zhenjiang 212100, China; 2 Department of Respiratory Medicine, the Affiliated Hospital of Jiangsu University, Zhenjiang 212001, China; 3 School of Optoelectronic Science and Engineering, University of Electronic Science and Technology of China, Chengdu 610054, China

**Keywords:** Lung-nodule detection, YOLOv5s, Omni-dimensional dynamic convolution, Lightweight, Wasserstein distance, Convolutional neural networks (CNNs)

## Abstract

**Background:**

Early and timely detection of pulmonary nodules and initiation treatment can substantially improve the survival rate of lung carcinoma. However, current detection methods based on convolutional neural networks (CNNs) cannot easily detect pulmonary nodules owing to low detection accuracy and the difficulty in detecting small-sized pulmonary nodules; meanwhile, more accurate CNN-based models are slow and require high hardware specifications.

**Objective:**

The aim of this study is to develop a detection model that achieves both high accuracy and real-time performance, ensuring effective and timely results.

**Methods:**

In this study, based on YOLOv5s, a concentrated-comprehensive convolution (C3_ODC) module with multidimensional attention is designed in the convolutional layer of the original backbone network for enhancing the feature-extraction capabilities of the model. Moreover, lightweight convolution is combined with weighted bidirectional feature pyramid networks (BiFPNs) to form a GS-BiFPN structure that enhances the fusion of multiscale features while reducing the number of model parameters. Finally, Focal Loss is combined with the normalized Wasserstein distance (NWD) to optimize the loss function. Focal loss focuses on carcinoma-positive samples to mitigate class imbalance, whereas the NWD enhances the detection performance of small lung nodules.

**Results:**

In comparison experiments against the YOLOv5s, the proposed model improved the average precision by 8.7% and reduced the number of parameters and floating-point operations by 5.4% and 8.2%, respectively, while achieving 116.7 frames per second.

**Conclusion:**

The proposed model balances high detection accuracy against real-time requirements.

## 1 INTRODUCTION

Early diagnosis and intervention are essential for improving lung cancer outcomes [1]. Computed tomography (CT) scans, X-ray chest radiography, and magnetic resonance imaging are among the current techniques for detecting and diagnosing lung nodules. Owing to their high resolution, sensitivity, and specificity, CT scans are especially useful and are commonly used in clinical practice. Traditional lung-nodule detection methods, which heavily rely on the experience and visual observation of medical professionals, are inefficient, error-prone, and delay the diagnosis. Advancements in computer and image-processing technologies have led to automated or semi-automated analysis of CT images, enabling the automatic identification and localization of lung nodules. This development has greatly improved the efficiency and accuracy of early lung cancer screening.

Deep learning-based medical image analysis methods have been widely applied in recent years. Most importantly, pulmonary nodules can now be detected by convolutional neural networks (CNNs) [2-5]. Among the CNN-based object detection methods are Faster Region-based CNN (Faster R-CNN) [6], You Only Look Once (YOLO) [7], and single-shot multibox detector (SSD) [8]. After training, these algorithms can automatically detect and localize lung nodules, largely improving the accuracy and reliability of early lung cancer screening and diagnosis. YOLO is recognized as an efficient, fast, and precise object detection method, but when applied to lung-nodule detection, it cannot efficiently detect small lung nodules. Therefore, YOLO predictions can lead to missed or misdiagnosed cases. Compared with detection algorithms such as Faster R-CNN, YOLO also tends to sacrifice accuracy for an enhanced detection speed. Many scholars have already applied the YOLO model to the detection of lung nodules, for example, Hongwu *et al*. [9] designed the YOLOv8-FE model with a size of only 2.3M, which meets the real-time requirement of deploying on the end of the mobile device, but the detection accuracy is only about 70%, and the problem of leakage and misdetection is serious. Ji *et al*. [10] proposed an improved YOLOv5-CASP model based on YOLOv5s, which improved the detection accuracy of lung nodules by 9 percentage points but doubled the model size and tripled the training time, which greatly affected the detection speed. The purpose of this paper is to improve the detection efficiency of small lung nodules and reduce the problem of misdetection and leakage, and at the same time, to improve the computational speed of the model to meet the requirements of real-time, so it is necessary to strike a balance between the detection accuracy and detection speed. Therefore, this paper proposes a lightweight lung-nodule detection method that integrates multidimensional attention based on the YOLOv5 algorithm. The main contributions of this paper are as follows:

1) Through progressive multiplication of different attention values along different dimensions (position, channel, filter, and kernel), multidimensional attention was introduced to the convolution layers of the original backbone network to form a concentrated-comprehensive convolution (C3_ODC) module with multidimensional attention. This approach provides the convolution operation with diverse multidimensional inputs, improving the performance of capturing rich contextual information and thereby enhancing the feature-extraction capability of the model.

2) In the network-feature fusion section, lightweight convolutions are combined with a weighted bidirectional feature pyramid network (BiFPN) to form a GS-BiFPN structure. This design reduces the number of parameters and computational burden, meeting the requirements of real-time detection. It also captures the multiscale feature information, enhancing the fusion of features at different scales.

3) Focal Loss and the normalized Wasserstein distance (NWD) are combined into the focal-normalized Wasserstein distance (F-NWD) loss function to optimize the model. Focal loss focuses on carcinoma-positive samples to mitigate sample imbalance, while the NWD enhances the model’s ability to detect small lung nodules.

The paper is organized as follows. Section 2 describes the research progress and existing methods of related work. Section 3 elaborates on our proposed method and its architecture. Section 4 shows the experimental procedure and experimental results, which are analyzed and discussed in comparison with other state-of-the-art models. Finally, our conclusions are drawn in Section 5.

## 2 MATERIALS AND METHODS

In early-stage lung cancer diagnosis, pulmonary nodules are detected by two main methods: traditional machine learning algorithms that rely on feature engineering and deep learning algorithms based on neural networks. Traditional machine learning methods use classification algorithms and manually designed features to identify pulmonary nodules. The identification process typically involves four steps: feature extraction, feature selection, classifier training, and detection and localization. Feature extraction isolates the manually designed features such as pixel intensity, texture, and shape information from lung CT images. Next, the most representative and discriminative features are selected to reduce the dimensionality and improve the algorithm's performance. In the classifier training step, the machine learning classifier is trained on the selected features. Finally, the classifier detects and localizes pulmonary nodules in image blocks at different positions in new CT images. Methods based on machine learning heavily rely on feature extraction and manual intervention., Shariaty *et al*. [11] designed a reconstruction technique and an active contour method that determines candidate nodules and then detects nodules using support vector machines. Paing *et al*. [12] proposed a three-dimensional (3D) chain coding algorithm that detects nodules attached to boundaries, providing more detailed nodule information with a reduced workload, thus improving the accuracy of detection. Although machine learning-based methods have achieved some success in early lung-nodule detection, their performance is limited by the manually designed features and the limited capabilities of classifiers. Therefore, they cannot meet the demands of high accuracy and generalization. Unlike machine learning approaches, deep learning methods excellently capture the complex patterns and features within images, offering higher accuracy and robustness. Based on whether they generate candidate regions, lung-nodule detection algorithms based on deep learning can be divided into two-stage and one-stage detection algorithms. Two-stage detection algorithms such as Faster R-CNN and Mask R-CNN [13] first generate a set of candidate regions containing pulmonary nodules and then perform false-positive removal and localization on these regions. Although these methods are accurate and adaptable to pulmonary nodule detection tasks, they are computationally complex and resource-intensive. Furthermore, to enhance the sensitivity of Faster R-CNN, Nguyen *et al*. [14] proposed an approach that automatically learns anchor-box configurations based on actual nodule sizes. Employing a residual network, they also reduced the number of false-positive outputs. Liao *et al*. [15] incorporated an attention mechanism into the Mask R-CNN pulmonary nodule detection algorithm. The improved algorithm enhances the contextual information through a multiscale pyramid while the attention models extract the rich channel and spatial information. Single-stage detection algorithms (such as YOLO and SSD) employ a single-neuron network model that directly detects objects on the entire image, negating the need for additional region-proposal generation steps. Although this approach increases the detection speed, it lowers the detection accuracy. Additionally, to improve the sensitivity of pulmonary nodule detection in the YOLO network, Han *et al*. [16] applied an attention mechanism to feature extraction and fused multiscale bidirectional feature pyramids. Ma *et al*. [17] presented a group-attention-based SSD model that efficiently extracts the contextual information between consecutive slices using group convolutions, thereby improving the detection performance.

The YOLO model is a popular choice in object detection studies because it converts object detection tasks into regression problems, which enables the optimization of end-to-end detection, making the detection rapid and efficient. The first YOLO algorithm, YOLOv1, proposed by Redmon *et al*. [18], unifies the object detection process by simultaneously detecting all bounding boxes, losing some accuracy for real-time performance. Later iterations of YOLO (YOLOv2–YOLO-v8) gradually enhanced the detection accuracy of YOLO toward the ultimate goal: simultaneously meeting the requirements of real-time processing and high precision. However, in medical CT image detection, 3D structures must be sliced into two-dimensional (2D) images for detection, which loses some of the feature information and complicates the detection process. In recent years, researchers have incorporated attention mechanisms into the YOLO network to improve its accuracy and generalization in pulmonary nodule detection. Yang *et al*. [19] introduced coordinate attention to the YOLO model, enabling the capture of position awareness, orientation awareness, and cross-channel information of pulmonary nodules. Liu *et al*. [20] utilized a deformable attention module for selective learning and filtering of shallow features to help the model capture finer contextual information and improve sensitivity to key details. Other researchers have improved the convolutional layer of the target detection model to enhance detection performance or reduce model complexity. Mei *et al*. [21] improved the feature extraction and semantic representation of the backbone network using a deep over-parameterized convolution layer (DO-Conv) and enhanced the feature pyramid structure to better detect small targets. Guo *et al*. [22] combined cascade R-CNN and feature pyramid networks into a multiscale detection model that fuses the features in the upper and lower layers, obtaining rich contextual information that improves the detection accuracy of small-sized nodules. Although individual improvements can modestly enhance the model performance, they often only marginally increase the detection accuracy or substantially increase the computational complexity and detection time. The present paper combines multiple improvement techniques into novel feature-extraction modules with attention-integrated convolution layers. It also enhances the bottom–up feature fusion channels and improves the lightweight convolutional layers. These enhancements boost the detection capability and generalization of the model without compromising its real-time performance. Finally, the paper improves the loss function to enhance the detection of small pulmonary nodules.

## 3 RESULTS

At present, YOLOv5 is the most widely used and best-generalized feature-detection model. The YOLOv5 series is divided into four models (s, m, l, and x) with different model sizes. In addition, to balance speed and accuracy, this paper enhances the YOLOv5s model. The improved lung-nodule detection model is depicted in Fig. (**1**). The improved model consists of three parts: the backbone network, the feature fusion network, and the detection head. The C3_ODC module is first constructed using full-dimensional convolution containing multi-dimensional attention in the convolutional layer of the original backbone network, which incrementally multiplies the convolution with different attention along the dimensions of position, channel, filter, and kernel, making the convolution operation differentiated in each dimension with respect to the inputs, and providing a better performance for capturing rich contextual information, thus enhancing the feature extraction capability of the model. Then, a two-way weighted feature pyramid network is used to improve the ability to capture multi-scale feature information and enhance multi-scale feature fusion while reducing the computational complexity of the model and improving detection speed. Finally, the model is optimized by combining focus loss and normalized Wasserstein distance weighting fused into a new loss function. The focus loss can focus on the positive samples and solve the problem of positive and negative sample imbalance; the normalized Wasserstein distance can improve the detection effect of the model on lung nodules.

### 3.1 The C3_ODC Module

The convolution module, which extracts the input features, is the basic module of a CNN. However, standard convolution can only learn fixed convolutional kernels and might fail to extract information on important features. An attention mechanism mimics human visual attention, thereby improving a model’s ability to identify significant regions and express features. The dynamic convolution integrates attention into CNNs by learning a combination of *N* convolutional kernels and applying attention weights. Unlike standard convolution, dynamic convolution adaptively adjusts the shape and weights of the convolutional kernels as follows:


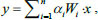
(1)

where *x* and *y* are the input and output data, respectively, *W_i_* denotes the *i*-th convolutional kernel, and the weight *α_i_* are calculated based on the input features. The dynamic convolution expressed by Eq. (**1**) includes the convolutional kernel and an attention function. Current dynamic convolution methods such as CondConv [21] and DyConv [22] do not comprehensively explore the convolution kernels. CondConv learns and calculates a linear combination of specialized convolution kernels prior to convolution operations, whereas DyConv dynamically aggregates multiple parallel convolution kernels based on attention and dynamically adjusts the weight of each kernel. Both methods focus on a single dimension of information, namely, the number of convolution kernels, neglecting the spatial size, input-channel count, output-channel count, and other dimensions. Additionally, both methods substantially increase the number of convolution-kernel parameters.

ODConv [23] is a generalized dynamic convolution combining the concepts of CondConv and DyConv. Additionally, using multidimensional attention obtained through parallel strategies, it captures the complementary information across four convolution-kernel dimensions as follows:


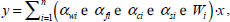
 (2)

where *α_wi_*, *α_fi_*, *α_ci_*, and *α_si_* represent the attention weights along the convolution-kernel number, output channel, input channel, and convolution-kernel space size, respectively. As shown in Fig. (**2**), the input feature map X is globally averaged pooling to create a feature vector matching the input-channel length. A fully connected layer maps this vector to a lower-dimensional space. The rectified linear unit (Relu) activates four branches with various outputs, which are then normalized by the Sigmoid function to generate attention weights across the four dimensions. In principle, attention along various convolutional dimensions (position, channel, filter, and kernel) allows the convolution operation to adapt to differences among different inputs, enabling better capture of the contextual information. Therefore, ODConv achieves higher feature-extraction capability than CondConv and DyConv while using fewer convolution kernels.

This paper exploits the advantage of portability, which enables the replacement of Convs of the C3 module with ODConv in yolov5, thereby enhancing the feature-extraction capability of the backbone network. The new C3_ODC module is depicted in Fig. (**3**).

When detecting lung nodules using YOLO, the conversion of lung CT images into 2D slices is prone to information loss and the presence of smaller-sized lung nodules that are difficult to detect. The optimized YOLOv5s model using the C3_ODC module acquires rich contextual information between slices to improve the detection precision and robustness of the model.

### 3.2 GS-BiFPN Neck Network

**Fig. (4a-c):**
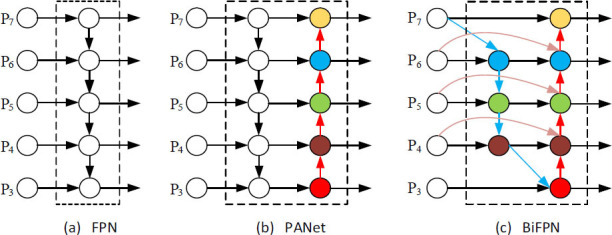
Structures of feature pyramid network (FPN), path aggregation network (PANet), and bidirectional FPN (BiFPN).

The neck network, which links the feature processing to information fusion in target detection, is usually configured as a feature pyramid structure that fuses low- and high-resolution feature maps and helps to maintain the integrity of semantic information. The first feature pyramid network (FPN) [24] was proposed by Lin *et al*. As shown in Fig. (**4a**), the FPN establishes a top-down feature fusion path with lateral connections that fuse the high- and low-level features, allowing the network to simultaneously focus on feature information at different scales. However, the traditional FPN is limited by the unidirectionality of the information transfer path. Liu *et al*. enhanced the FPN with a path aggregation network (PANet) [25] (Fig. **4b**), which establishes a bidirectional feature fusion path. Although PANet improves the integration of high-level semantics and low-level details, it determines nonuniform contributions of shallow and deep features if the input features have different resolutions. To address this issue, Tan *et al*. proposed the weighted BiFPN [26] and the structure shown in Fig. (**4c**). This network integrates the features of different levels and a two-way information transmission mechanism to achieve efficient and accurate multiscale target detection.

In this paper, the BiFPN network replaces the FPN and PANet networks in YOLOv5 to improve the efficiency of lung-nodule detection. The functionality of BiFPN is twofold: efficient bidirectional cross-scale connectivity and weighted feature map fusion. BiFPN amends PANet as follows.

Nodes with single-input edges, which contribute little to the feature fusion, are removed without noticeably affecting the network. Input and output nodes in the same layer are connected by an additional edge for enhanced feature fusion. Each bidirectional path (top–down and bottom–up) is processed as a feature network layer through multiple iterations to deepen the feature fusion. BiFPN employs a straightforward and efficient weighted feature fusion mechanism (Fig. **4c**). As an example, the output of the P6 layer is calculated as follows:


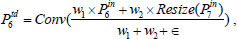
(3)


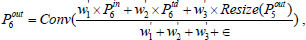
(4)

where *Resize* is a downsampling or upsampling operation, and *w* is a learned parameter.

Moreover, to reduce the complexity of the model and effectively balance the speed and accuracy, the original ordinary convolutional layer of the YOLOv5 neck is replaced with a lightweight GSConv convolutional layer [27]. Experimentally, it was verified that using GSConv in the backbone network leads to loss of semantic information as the network layer deepens, but using GSConv in the neck reduces the redundant information and preserves the inter-channel connectivity to the largest possible extent. GSConv performs a downsampling of ordinary convolution followed by a deep convolution using DWConv and then splices the results of both convolutions. Finally, a shuffle-blending operation integrates the information of the ordinary and DWConv convolutions. The structure of GSConv is depicted in Fig. (**5**).

### 3.3 Improvement of the Loss Function

The loss function in YOLOv5 is the complete intersection over union (CIOU) [28], which is very sensitive to deviations in the position of small objects. This sensitivity can largely degrade the detection performance, especially in images with numerous small-sized pulmonary nodules. Furthermore, as the pulmonary nodules occupy only a small portion of lung images, the classes are severely imbalanced. To address these challenges, this paper proposes the F-NWD loss function, which combines the focal loss [29] and NWD [30-32] in the optimization process.

NWD is lowly sensitive to the sizes of different objects and is suitable for measuring similarities between small objects. The NWD loss models bounding boxes as 2D Gaussian distributions and measure their similarity as the Wasserstein distance between them. The foreground and background pixels within the bracketing boxes of small targets tend to be distributed in the center and at the edges, respectively. To better weight each pixel within the bracketing box, the bracketing box can be modeled as a 2D Gaussian distribution as follows:


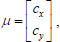
(5)


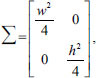
(6)

where *C_x_*, *C_y_*, *w*, and *h* denote the center coordinates, width, and height of the horizontal enclosing frame, respectively.

Borrowed from optimal transmission theory, the Wasserstein distance defines the distance between two distributions, as mentioned above. Using the normalized exponent, we obtain a new metric with the second-order Wasserstein distance


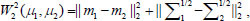
 (7)

which is normalized as follows:


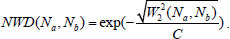
(8)

In Eq. (**7**), *µ*_1_ and *µ*_2_ denote two Gaussian distributions with means *m*_1_ and *m*_2_, respectively. *∑* denote the square roots of the covariance matrices of *µ*_1_ and *µ*_2_, respectively, || ||_2_ denotes the Euclidean distance, *NWD*(*N_a_, N_b_*) denotes the NWD, and *C* denotes a constant, which is dependent on the dataset and computational parameters.

In lung nodule detection, the positive samples (predicted bounding boxes containing lung nodules) occupy a small portion of all lung images; the majority of regions are negative samples with no lung nodules. The overwhelming ratio of negative to positive samples biases the prediction model toward the negative class, leading to a high false-positive rate and poor generalization. Focal Loss mitigates the class imbalance with a dynamic scaling factor that quickly directs the model’s attention to challenging samples during training. This factor is defined as


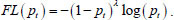
 (9)

The term (1*-p_t_*)*^λ^* in Eq. (**9**) is the modulation factor, which reduces the loss contribution of easy-to-score samples and increases the loss proportion of difficult-to-score samples, thus moving the model optimization toward a better outcome.

The combined Focal Loss and NWD function can effectively resolve the sample imbalance and size insensitivity in lung-nodule detection. It is formulated as


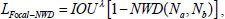
 (10)

where *NWD*(*N_a_*, *N_b_*) denotes the NWD and *IOU^λ^* is the weight on the sample loss, which balances the effects of high-quality and low-quality anchor boxes on the detection results and helps to improve the regression accuracy.

## 4 DISCUSSION

### 4.1 Dataset and Pre-processing

Evaluation experiments were performed on a subset of the world’s largest publicly available lung-nodule dataset (LIDC–IDRI). The LUNA16 dataset of LIDC–IDRI contains 1186 lung nodules in 888 lung CT images. The complete information of the CT images in the LUNA16 dataset is provided in two file formats: “.mhd” and “.raw,” with each CT scan containing a varying number of 2D axial slices. The annotations of each CT image are recorded in the “annotations.csv” file, which includes information such as the coordinates and diameters of the lung nodules.

The lung-nodule samples from LUNA16 were randomly assigned to training or test sets at a ratio of 8:2. To improve the detection accuracy, interferences of impurities such as bones and blood vessels were removed by extracting the lung parenchyma *via* a segmentation process. First, the lung CT images were denoised, and the density range was adjusted. The lungs and background were then separated by an image binarization process. After eliminating the noise and voids through morphological operations, the connected regions were marked to determine the lung boundaries. Finally, a mask of the lung parenchyma was generated. The segmented image is shown in Fig. (**6**).

### 4.2 Experimental Environment and Parameter Settings

Table **1** specifies the hardware environment of the experiments.

The parameters were set as follows: the size of preprocessed input images = 640 × 640; convolution kernel except C3_ODC module = 3 × 3; batch size = 8; initial learning rate = 0.01. The optimizer was stochastic gradient descent with momentum and weight decay values of 0.937 and 0.0005, respectively, and training was performed over 200 epochs.

### 4.3 Evaluation Indicators

The proposed algorithm was dually evaluated for its detection accuracy and detection speed. The performance measures include the precision, recall, mean average precision (*mAP*), size of the model parameters (Params), floating-point operations (FLOPs), and frames per second (FPS). The precision primarily reflects the ability of the model to handle false positives, where a higher precision (*P*) indicates a lower false-positive rate. The recall (or recall rate) is the detection rate of all true-positive lung nodules, where a higher recall (*R*) implies a lower false-negative rate. In the context of the present study, which attempts to detect lung nodules only, the *mAP* is equivalent to the average precision derived from the *P–R* curve. The *P*, *R*, and *mAP* are respectively determined as follows:


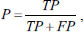
(11)


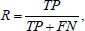
(12)


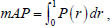
 (13)

where *TP*, *FP*, and *FN* represent the numbers of true positives, false positives, and false negatives, respectively, and *P*(*r*) signifies the *P–R* curve.

Params refer to model parameters and reflect their size. FLOPs quantify the computational load, signifying the algorithmic complexity and detection speed. FPS represents the number of images processed per second, where higher values indicate faster processing.

### 4.4 Experimental Results and Analyses

#### 4.4.1 Visualization of Model Training

Fig. (**7**) illustrates the change in loss during the training and validation of the model. In the initial stage, both the training loss and the validation loss decrease rapidly, indicating that the model learns a lot of useful information in this stage and improves its fitting ability to the training data. With the increase of epoch, both curves gradually level off and are very close to each other, indicating that the model has good generalization performance.

#### 4.4.2 Ablation Experiments

##### 4.4.2.1 F-NWD Loss Function Weight Analysis

For the improved loss function, Focal loss and NWD are experimented with different weights, and the loss image is calculated as shown in Fig. (**8**).

Fig. (**8**) shows the loss of the improved model stabilizes after 200 epochs, and the single NWD loss function cannot solve the problem of positive and negative sample imbalance. However, because the NWD loss function can also accurately calculate the positional deviation of tiny targets and improve the detection ability of small lung nodules, the experimental improvement of the loss function in this chapter uses Focal loss accounted for 0.1 and NWD accounted for 0.9 of the weights for model training, and the results show that at this time the loss function value reaches the lowest, and achieves the best results.

##### 4.4.2.2 Performance Comparison of Models based on Different Attention Mechanisms

In order to verify the effectiveness of the C3_ODC module designed in this paper for model performance enhancement, several attention mechanisms are selected for comparative experiments, including Spatial Attention (SA), Efficient Channel Attention (ECA), Convolutional Block Attention (CBAM) and Coordinate Attention (CA). The comparison experiments here are only different in the attention of composing the new feature extraction block; all other parts are the same, and the comparison results are shown in Table **2**.

Comparison results show that C3_SA and C3_ECA achieve good results in terms of detection speed, but their focus on only one-sided information leads to a small improvement in detection performance.C3_ODC improves detection accuracy by 3 percentage points compared to C3_CBAM and C3_CA, which is due to the fact that the multidimensional attention mechanism is able to capture features from different dimensions, which helps the model to understand the data more comprehensively, thus obtaining richer feature representations, although the number of parameters and computation are increased, the improvement is acceptable compared to the detection accuracy.

##### 4.4.2.3 Ablation Experiments

Furthermore, to further validate the impact of the improved module on lung-nodule detection, we performed ablation experiments on the three improved parts on the same dataset under the same parameter settings as the benchmark (YOLOV5s) model. The results are shown in Table **3**.

As indicated in Table **3**, our method achieves higher accuracy and detection speed than the original model. More specifically, the proposed model increased the *mAP* and FPS by 8.7% and 5.1%, respectively, and reduced Params and FLOPs by 5.4% and 8.2%, respectively, from those of YOLOv5s. Replacing the C3 module in the backbone network with the C3_ODConv module and applying the F-NWD loss function substantially improved the detection accuracy. This improvement is attributed to the adaptive shape and weight changes of the convolution kernels and the multidimensional attention mechanism in ODConv. When integrated into the C3 module, the C3_ODConv module captures more contextual information than the original module, thereby enhancing the feature-extraction capability of the backbone network. Meanwhile, the improved loss function F-NWD focuses the feature maps on the target region, thus improving the detection of positive samples, whereas the normalized Wasserstein distance enables easy detection of small-sized lung nodules. The improved neck structure GS-BiFPN, achieved through feature weighting and multipath multiscale feature fusion of the important features, further improves the detection accuracy. More importantly, the lightweight convolution module GSConv substantially increases the detection speed of the model.

Additionally, to visually demonstrate the detection effect of the improved model, representative lung-nodule images were selected from the LUNA16 dataset after segmenting the lung parenchyma. These images included multiple, isolated, tiny, near-vessel, or near-lung-wall nodules. The detection results of the proposed model and the original YOLOv5s model are compared in Fig. (**9**).

Fig. (**9**) shows that when detecting lung images with multiple nodules, YOLOv5s falsely detected an extra lung nodule, resulting in a false-positive prediction. In contrast, the proposed model accurately identified the nodules, indicating its higher accuracy than YOLOv5s. YOLOv5s also missed some of the extremely small nodules, which were detected by the proposed model. This improvement was attributed to the NWD in the proposed loss function, which improved the small lung-nodule detection. Furthermore, although YOLOv5s and the proposed model accurately detected isolated nodules, near-vascular nodules, and near-pulmonary wall nodules, the proposed model obtained a higher detection score than YOLOv5s. Overall, the improved model reduces the number of false positives, improves the detection accuracy, and achieves superior detection results.

#### 4.4.3 Comparative Experiments

This research aimed to balance the tradeoff between detection speed and detection accuracy of a lung-nodule detection model in real-time. Moreover, to prove the effectiveness and superiority of the detection model, the performance of the model was compared with those of other mainstream lung-nodule detection models (Faster R-CNN, SSD, and the YOLO series). The *mAP*, Params, FLOPs, and FPS parameters of the compared models on the LUNA16 dataset are listed in Table **4**.

Compared with the two-stage lung-nodule detection model Faster R-CNN, the developed model raised the mAP value by 12%, reduced the number of parameters and computations by 74.06M and 286.9G, respectively, and increased the detection frame rate by 103.4 FPS. The developed model achieved the highest average detection accuracy for the one-stage detection algorithms, increasing the mAP by more than 6% from those of YOLOv5s, YOLOv7-Tiny, and YOLOv9s with a similar number of parameters. The number of parameters in the developed model only slightly exceeded that in YOLOv7-Tiny and is much smaller than that in RT-DETR, YOLOv5l, and YOLOv7. The detection frame rate of the developed model (116.7 FPS) was 75.3 FPS higher than that of YOLOv5l, which has the second-highest average detection accuracy. The comparative analysis demonstrates the superior detection accuracy, model size, and inference speed of the developed model, confirming its ability to balance detection accuracy with speed optimization.

## CONCLUSION

This paper introduces a lightweight lung-nodule detection model tailored for hospitals with abundant medical data but constrained hardware. The aim is to maximize both the accuracy and efficiency of detection. The model is an adaptation of YOLOv5s with various improvements, including a constructed feature-extraction module, an improved convolutional layer structure, a replaced BiFPN structure, and an optimized loss function, for improving the detection accuracy and detection speed of the model.”. The model was evaluated on the LUNA16 lung-nodule dataset using multiple metrics (*mAP*, Params, and FPS) and its performance was compared with those of other target detection models. The experimental results show that the improved model proposed in this paper achieves a detection accuracy of 92.8%, a parameter count of 6.64 M, a computational complexity of 14.6 G, and a detection speed of 116.7. Both the detection speed and detection accuracy are improved to different degrees compared with other state-of-the-art detection models, which is expected to promote a new step forward in the field of deep learning technology in the detection of lung nodules and to help doctors make early lung nodule diagnosis. In further research, the same lung-nodule feature information should be extracted from multiple 2D images to further optimize the model and improve its lung-nodule detection ability. The dataset should also be expanded with samples of novel variants of lung nodules to improve the robustness of the model.

## Figures and Tables

**Fig. (1):**
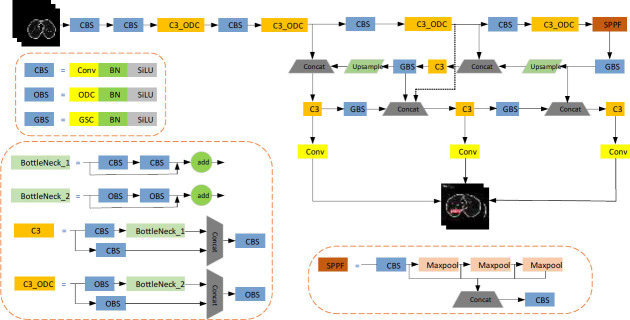
General structure of the improved algorithm.

**Fig. (2):**
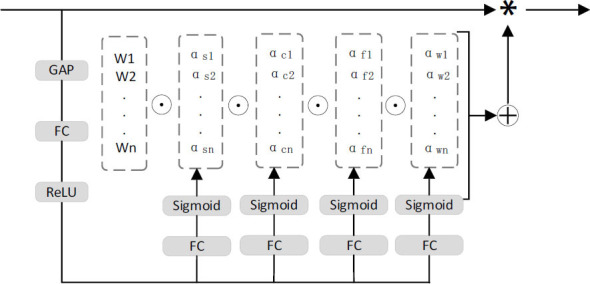
Structure of omnidimensional dynamic convolution.

**Fig. (3):**
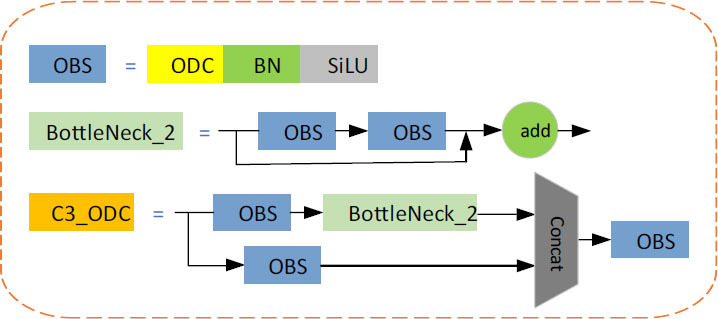
C3_ODC structure.

**Fig. (5):**
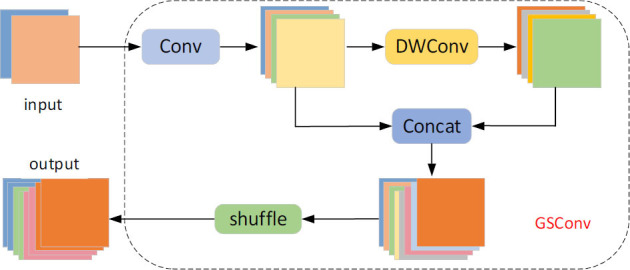
GSConv structure.

**Fig. (6):**
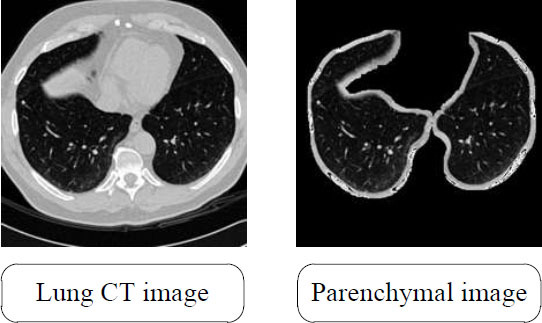
CT images of lungs before segmentation (left) and with parenchymal image (right).

**Fig. (7):**
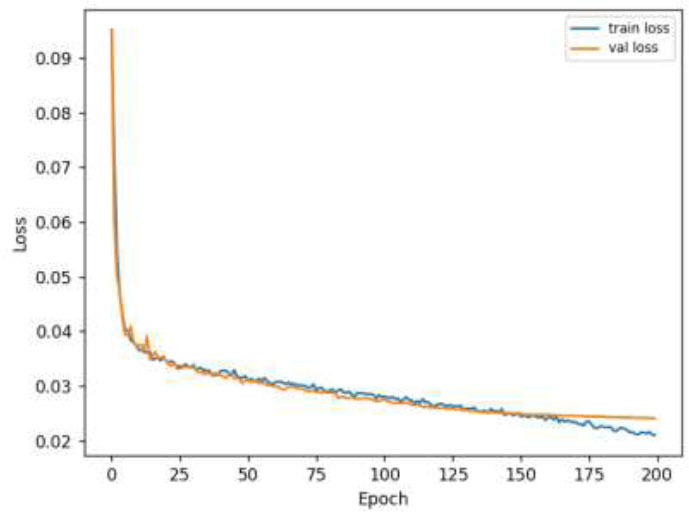
Model loss change curve.

**Fig. (8):**
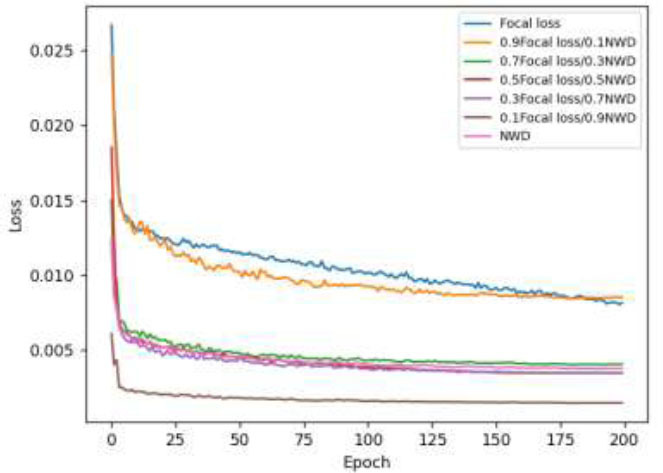
Improved Focal loss and NWD loss curves of the model at different weights.

**Fig. (9):**
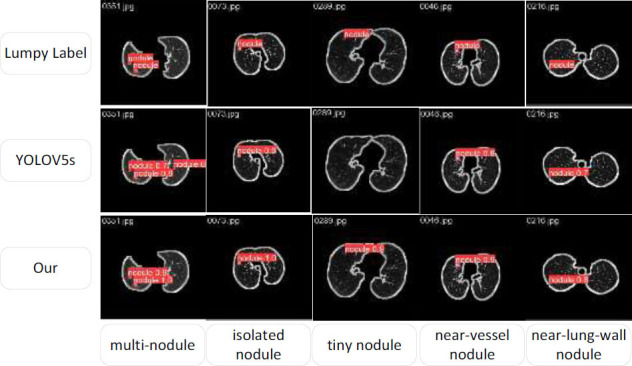
Comparison of lung parenchyma detection results of YOLOv5s and the proposed model.

**Table 1:** Hardware configuration of the validation experiment.

Experimental platforms	Environmental Configuration
Operating system	Win10
Deep learning frameworks	Pytorch-gpu1.13.1
Programming language	Python3.7
CPU	Intel(R) Core(TM) i5-12600KF
GPU	NVIDIA GeForce RTX 3070 Ti
RAM	32GB
CUDA	NVDIA CUDA 11.7

**Table 2:** Comparison of model performance using different attention.

Attention	mAP (%)	Params (M)	FLOPs (G)	FPS
C3	87.1	5.69	6.18	168.63
C3_SA	87.5	5.67	6.10	170.61
C3_ECA	87.9	5.67	6.45	173.30
C3_CBAM	89.2	6.49	6.96	162.67
C3_CA	89.8	6.43	6.93	171.01
C3_ODC	92.8	6.64	7.11	169.50

**Table 3:** Results of ablation experiments.

C3_ODConv	GS-BiFPN	F-NWD	mAP (%)	Params (M)	FLOPs (G)	FPS
-	-	-	84.1	7.02	15.9	111
√	-	-	90.9	7.15	8.7	106
-	√	-	86.5	6.34	15.4	138.8
-	-	√	89.8	7.02	15.9	111.2
√	√	-	91.6	6.64	14.6	117.3
√	-	√	92.1	7.16	8.9	106.5
-	√	√	91.1	6.41	15.8	136.9
√	√	√	92.8	6.64	14.6	116.7

**Table 4:** Performance comparison of different detection models.

Models	mAP (%)	Params (M)	FLOPs (G)	FPS
Faster R-CNN	80.8	80.70	301.5	13.3
SSD	78.7	22.33	92.6	42.1
RT-DETR	87.6	20.57	61.8	53.9
YOLOv3	82.9	60.47	124.7	36.8
YOLOv5s	84.1	7.02	15.9	111
YOLOv5m	87.2	21.21	48.6	57
YOLOv5l	90.5	46.86	109.3	41.4
YOLOv7	88.4	37.25	105.2	42.9
YOLOv7-Tiny	74.7	6.03	13.2	127.3
YOLOv8s	86.1	11.10	28.7	71.4
YOLOv9s	86.5	7.11	26.7	99.8
Our	92.8	6.64	14.6	116.7

## Data Availability

The original dataset is available at https://luna16.grand-challenge.org/, but we edited it to be convenient for our task. The edited dataset has not been available online yet.

## LIST OF ABBREVIATIONS

F-NWD: Focal-Normalized Wasserstein Distance
CT: Computed Tomography
NWD: Normalized Wasserstein Distance
BiFPNs: Bidirectional Feature Pyramid Networks
CNNs: Convolutional Neural Networks

## References

[1] Sung H., Ferlay J., Siegel R.L., Laversanne M., Soerjomataram I., Jemal A., Bray F. (2021). Global cancer statistics 2020: Globocan estimates of incidence and mortality worldwide for 36 cancers in 185 countries.. CA Cancer J. Clin..

[2] Zhang W., Wang X., Li X., Chen J. (2018). 3D skeletonization feature based computer-aided detection system for pulmonary nodules in CT datasets.. Comput. Biol. Med..

[3] Kaya A., Can A.B. (2015). A weighted rule based method for predicting malignancy of pulmonary nodules by nodule characteristics.. J. Biomed. Inform..

[4] Chen H., Xiong W., Wu J., Zhuang Q., Yu G. (2020). Decision-making model based on ensemble method in auxiliary medical system for non-small cell lung cancer.. IEEE Access.

[5] Wu J., Tan Y., Chen Z., Zhao M. (2018). Decision based on big data research for non-small cell lung cancer in medical artificial system in developing country.. Comput. Methods Prog. Biomed..

[6] Ren S., He K., Girshick R., Sun J. (2017). Faster R-CNN: Towards real-time object detection with region proposal networks.. IEEE Trans. Pattern Anal. Mach. Intell..

[7] Jiang P., Ergu D., Liu F., Cai Y., Ma B. (2022). A review of Yolo algorithm developments.. Procedia Comput. Sci..

[8] Liu W., Anguelov D., Erhan D., Szegedy C., Reed S., Fu C.Y., Berg A.C. (2016). SSD: Single Shot MultiBox Detector. Computer Vision–ECCV 2016. Lecture Notes in Computer Science.

[9] Ji Z., Wu Y., Zeng X., An Y., Zhao L., Wang Z., Ganchev I. (2023). Lung nodule detection in medical images based on improved Yolov5s.. IEEE Access.

[10] Hongwu Q., Xinyu W., Fei X. (2024). Pulmonary nodule detection algorithm based on lightweight improved YOLOv8 network model.. Bull. Pacific State Uni..

[11] Shariaty F., Davydov V.V., Yushkova V.V., Glinushkin A.P., Rud V.Y. (2019). Automated pulmonary nodule detection system in computed tomography images based on Active-contour and SVM classification algorithm.. J. Phys. Conf. Ser..

[12] Paing M.P., Hamamoto K., Tungjitkusolmun S., Visitsattapongse S., Pintavirooj C. (2020). Automatic detection of pulmonary nodules using three-dimensional chain coding and optimized random forest.. Appl. Sci..

[13] He K., Gkioxari G., Dollár P., Girshick R. (2017). Mask r-cnn.. arXiv:1703.06870.

[14] Nguyen C.C., Tran G.S., Nguyen V.T., Burie J.C., Nghiem T.P. (2021). Pulmonary nodule detection based on faster R-CNN with adaptive anchor box.. IEEE Access.

[15] Liao J., Li W., Du L.Y. Detection of pulmonary nodule lesions based on improved Mask R-CNN algorithm.. International Conference on Artificial Intelligence and Intelligent Information Processing (AIIIP 2022).

[16] Han L., Li F., Yu H., Xia K., Xin Q., Zou X. (2023). BiRPN-YOLOvX: A weighted bidirectional recursive feature pyramid algorithm for lung nodule detection.. J. XRay Sci. Technol..

[17] Ma J., Li X., Li H., Menze B.H., Liang S., Zhang R., Zheng W.S. (2019). group-attention single-shot detector (GA-SSD): Finding pulmonary nodules in large-scale CT images.. Proceed. Mach. Learn. Res..

[18] Redmon J., Divvala S., Girshick R., Farhadi A. (2016). You only look once: Unified, real-time object detection.. Proceedings of the IEEE conference on computer vision and pattern recognition.

[19] Yang L.I., Shiqi G.A.O. (2022). Lung nodule detection system based on data augmentation and attention mechanism.. J. Beijing Uni. Posts. Telecommun..

[20] Liu Y., Ao Y. (2024). Deformable attention mechanism-based YOLOv7 structure for lung nodule detection.. J. Supercomput..

[21] Mei S., Jiang H.Q., Ma L. (2021). YOLO-lung: A practical detector based on imporved YOLOv4 for pulmonary nodule detection.. 2021 14th International Congress on Image and Signal Processing, BioMedical Engineering and Informatics (CISP-BMEI).

[22] Guo N., Bai Z. (2021). Multi-scale pulmonary nodule detection by fusion of cascade R-CNN and FPN.. 2021 International Conference on Computer Communication and Artificial Intelligence (CCAI).

[23] Yang B., Bender G., Le Q.V., Ngiam J. (2019). Condconv: Conditionally parameterized convolutions for efficient inference.. Proceedings of the 33rd International Conference on Neural Information Processing Systems.

[24] Chen Y., Dai X., Liu M., Chen D., Yuan L., Liu Z. (2020). Dynamic convolution: Attention over convolution kernels.. Proceedings of the IEEE/CVF conference on computer vision and pattern recognition..

[25] Li C., Zhou A., Yao A. (2022). Omni-dimensional dynamic convolutio.. arXiv:2209.07947.

[26] Lin T.Y., Dollár P., Girshick R., He K., Hariharan B., Belongie S. (2017). Feature pyramid networks for object detection.. Proceedings of the IEEE conference on computer vision and pattern recognition.

[27] Liu S., Qi L., Qin H., Shi J., Jia J. (2018). Path aggregation network for instance segmentation.. Proceedings of the IEEE conference on computer vision and pattern recognition.

[28] Tan M., Pang R., Le Q.V. (2020). Efficient Det: Scalable and efficient object detection.. ArXiv:1911.09070, 2020..

[29] Li H., Li J., Wei H., Liu Z., Zhan Z., Ren Q. (2022). Slim-neck by GSConv: A better design paradigm of detector architectures for autonomous vehicles.. https://arxiv.org/vc/arxiv/papers/2206/2206.02424v1.pdf.

[30] Zheng Z., Wang P., Liu W., Li J., Ye R., Ren D. (2020). Distance-IOU loss: Faster and better learning for bounding box regression.. Proc. Conf. AAAI Artif. Intell..

[31] Zhang Y.F., Ren W., Zhang Z., Jia Z., Wang L., Tan T. (2022). Focal and efficient IOU loss for accurate bounding box regression.. Neurocomputing.

[32] Wang J., Xu C., Yang W., Yu L. (2021). A normalized Gaussian Wasserstein distance for tiny object detection.. arXiv:2110.13389.

